# Supplementary material for: Cross-sectional associations of physical frailty with fall, multiple falls and fall-injury among older Indian adults: Findings from LASI, 2018
Source: PLoS One. 2022 Aug 12;17(8):e0272669. doi: 10.1371/journal.pone.0272669 (PMC9374260; doi:10.1371/journal.pone.0272669)
Supplement: Attachment — Submitted filename: Response file_Rev1.docx [file pone.0272669.s001.docx]

**Title: Cross-sectional associations of frailty with fall, multiple falls and fall-injury among older Indian adults: findings from LASI, 2018**

**Editor comments**

The manuscript has received encouraging reviews. Please address the issues of the reviewers and we will reconsider the manuscript.

**Response: Dear Editor, the authors are thankful to you and both the reviewers for pointing out the merits of the study and recommending the paper to be published in the journal after incorporating the comments.**

**Review Comments to the Author**

**Reviewer #1:**

Thank you for the opportunity to review this manuscript. It is well written and on an important topic. My suggestions for revisions include:

Abstract:

1.) Need to define the term SRH in the abstract

**Response: The SRH is defined now at its first use.**

Background

2.) Please state your hypothesis in the background

**Response: The hypotheses of the study are mentioned now in the background.**

3.) In the conceptual framework you need to define the acronym MPCE

**Response: The MPCE is defined in the revised framework.**

4.) Please give a more detailed account of the tests used in your statistical analysis

**Response: More details are provided for the tests used in the statistical analyses.**

5.) SRH needs to be defined in the text

**Response: SRH is defined at first use now.**

**Reviewer #2:**

This is a well conducted study in a large population. This study highlights that frailty is not only a clinical and social problem for aging western developed countries but likely a major problem for other eastern large countries as well. The authors paper is well written and data analysis and conclusions are sound.

**Response: Dear reviewer, many thanks for pointing out the merits of the study and the recommendation.**
